# Differential Role of the RAC1-Binding Proteins FAM49b (CYRI-B) and CYFIP1 in Platelets

**DOI:** 10.3390/cells13040299

**Published:** 2024-02-06

**Authors:** Dmitri Sisario, Markus Spindler, Katharina J. Ermer, Noah Grütz, Leo Nicolai, Florian Gaertner, Laura M. Machesky, Markus Bender

**Affiliations:** 1 Institute of Experimental Biomedicine–Chair I, University Hospital Würzburg, 97080 Würzburg, Germany; 2 Medizinische Klinik und Poliklinik I, University Hospital Ludwig, Maximilian University, 81377 Munich, Germanyf.gaertner@med.uni-muenchen.de (F.G.); 3 German Centre for Cardiovascular Research, Partner Site Munich Heart Alliance, 81377 Munich, Germany; 4 Department of Biochemistry, University of Cambridge, Sanger Building, 80 Tennis Court Road, Cambridge CB2 1GA, UK

**Keywords:** platelets, FAM49b, CYRI-B, RAC1, actin, CYFIP1, spreading, lamellipodia, migration, WAVE

## Abstract

Platelet function at vascular injury sites is tightly regulated through the actin cytoskeleton. The Wiskott–Aldrich syndrome protein-family verprolin-homologous protein (WAVE)-regulatory complex (WRC) activates lamellipodia formation via ARP2/3, initiated by GTP-bound RAC1 interacting with the WRC subunit CYFIP1. The protein FAM49b (Family of Unknown Function 49b), also known as CYRI-B (CYFIP-Related RAC Interactor B), has been found to interact with activated RAC1, leading to the negative regulation of the WRC in mammalian cells. To investigate the role of FAM49b in platelet function, we studied platelet-specific *Fam49b*^−/−^-, *Cyfip1*^−/−^-, and *Cyfip1*/*Fam49b*^−/−^-mice. Platelet counts and activation of *Fam49b*^−/−^ mice were comparable to those of control mice. On fully fibrinogen-coated surfaces, *Fam49b*^−/−^-platelets spread faster with an increased mean projected cell area than control platelets, whereas *Cyfip1*/*Fam49b*^−/−^-platelets did not form lamellipodia, phenocopying the *Cyfip1*^−/−^-platelets. However, *Fam49b*^−/−^-platelets often assumed a polarized shape and were more prone to migrate on fibrinogen-coated surfaces. On 2D structured micropatterns, however, *Fam49b*^−/−^-platelets displayed reduced spreading, whereas spreading of *Cyfip1*^−/−^- and *Cyfip1*/*Fam49b*^−/−^-platelets was enhanced. In summary, FAM49b contributes to the regulation of morphology and migration of spread platelets, but to exert its inhibitory effect on actin polymerization, the functional WAVE complex must be present.

## 1. Introduction

Platelets monitor vessel wall integrity and play a crucial role in maintaining hemostasis. Upon vessel wall injury, platelets interact with exposed extracellular matrix components, triggering their adhesion, activation, and aggregation [1]. Activated platelets, together with fibrin generated by the coagulation cascade, form a stable plug which seals the wound and minimizes blood loss. However, uncontrolled thrombus formation can lead to life-threatening cardiovascular and cerebrovascular diseases, posing a significant health risk [2]. Over the last decades, platelets have been increasingly shown to also have pivotal roles in other (patho)physiological processes, like inflammation, metastasis, immune and pathogen responses, and organ and barrier maintenance.

Platelets circulate within the bloodstream as disc-shaped cells, with their structure maintained by microtubules arranged in a peripheral ring formation and the cross-linked filamentous network of the actin cytoskeleton. Upon activation, platelets dynamically rearrange their cytoskeletal components. When human or mouse platelets bind in vitro to adhesive substrates, such as fibrinogen, they first form filopodial structures with parallel actin filaments and then a broad circular branched actin network, the lamellipodium [3,4,5]. It has been shown that the pentameric WASp family verprolin-homologous protein (WAVE) complex plays an essential role in the formation of lamellipodia [6]. The WAVE complex consists of five subunits, namely, WAVE, ABI1, NAP1, CYFIP1, and HSPC300. The complex remains inactive until the subunit CYFIP1 interacts with the active form of the small GTPase RAC1. The active WAVE complex then associates with ARP2/3, a pivotal step in actin filament branching, and, consequently, lamellipodia formation [7]. Platelets deficient for RAC1 [8], ARP2/3 [9], or CYFIP1 [10] do not form lamellipodial protrusions with a circumferential zone of orthogonally arrayed short actin filaments [3]. It was demonstrated that lamellipodia formation in platelets is not necessary for hemostasis and thrombus formation in vivo [10], but it plays a critical role in platelet migration for vascular surveillance [11].

Recently, FAM49 (family of unknown function 49), also known as CYRI (CYFIP-related RAC interactor), was identified as a negative regulator of the WAVE complex by Fort and colleagues, who demonstrated that FAM49 binds activated RAC1 via a domain of unknown function (DUF1394) which is shared with CYFIP [12]. Mammals express two isoforms of the FAM49 protein family, FAM49a (CYRI-A) and FAM49b (CYRI-B) [13], which share an amino acid sequence identity of approximately 80% [14]. FAM49b is ubiquitously expressed, while FAM49a has a more restricted expression pattern [15]. FAM49a was described as a key regulator of macropinosome formation and integrin internalization [14]. Knockdown or knockout of FAM49b in COS-7 or CHL-1 cells enhanced the formation of large and wide lamellipodia with increased RAC1 signaling and induced an accumulation of WAVE proteins in these cell structures. In contrast, inducible overexpression of FAM49b led to fractal lamellipodia and reduced WAVE2 recruitment and RAC1 activation [12]. Thus, FAM49b was suggested to be a local inhibitor of RAC1 activity, thereby limiting lamellipodia size [12]. Mass spectrometry revealed that the protein expression of FAM49b with about 5300 copies per mouse platelet is similar to CYFIP1 (about 6000 copies/mouse platelet) [16]. Both proteins could, therefore, compete in the same ratio for RAC1 binding and regulate RAC1 activity. However, the role of FAM49b in platelets is, so far, unknown.

In this study, we investigated the role of FAM49b in platelets by analyzing platelet-specific *Fam49b*^−/−^-, *Cyfip1*^−/−^-, and *Cyfip1*/*Fam49b*^−/−^-mice. We propose that FAM49b plays a role in the regulation of platelet spreading and migration, but CYFIP1 deficiency abolishes the inhibitory effect of FAM49b on the WAVE complex.

## 2. Materials and Methods

### 2.1. Mice

The mouse line *Fam49b (Cyri*-*b)*, C57BL/6N-Cyrib^tm1a(KOMP)Wtsi^/Tcp (EPD0741_3_C08), was made at The Centre for Phenogenomics as part of the International Mouse Phenotyping Consortium [17] from KOMP ES cells [18] using published protocols [19]. Flp excision to generate the tm1c (conditional) allele was performed by breeding a heterozygous male C57BL/6N-Cyrib^tm1a(KOMP)Wtsi^/Tcp with heterozygous C57BL/6N-Gt(ROSA)26Sor^tm2(CAG−flpo,−EYFP)Ics^/Tcp female followed by subsequent backcrossing to C57BL/6NCrl. Frozen sperm for the line was obtained from the Canadian Mouse Mutant Repository and rederived into the animal facility on a C57BL/6J using IVF by a standard protocol [20]. Mice containing the *Cyfip1* gene (*Cyfip1^fl^*^/*fl*^) flanked by loxP sites were generated as described previously [10]. Conditional knockout mice were intercrossed with mice carrying the Cre-recombinase under the Pf4 promoter [21] to generate platelet- and megakaryocyte-specific knockout mice. Mice are further referred to as *Fam49b*^−/−^-, *Cyfip1*^−/−^-, or *Cyfip1*/*Fam49b*^−/−^-mice. Female and male mice older than 6 weeks of age were used in this study. Methods were performed in accordance with the relevant guidelines and regulations of the district government of Lower Franconia (Bezirksregierung Unterfranken).

### 2.2. Flow Cytometry

To determine platelet glycoprotein expression, whole blood was collected in heparin (20 U/mL) and further diluted, 1:20, in Tyrode’s HEPES buffer. Next, samples were incubated with fluorophore-labeled antibodies for 15 min at room temperature (RT) before analysis on an FACSCelesta (BD Biosciences, Heidelberg, Germany). To study platelet activation, blood samples were washed with Tyrode’s HEPES buffer, finally incubated in Tyrode’s HEPES buffer with 2 mM Ca^2+^ for 15 min with the corresponding agonist, and stained with fluorophore-labeled antibodies for active αIIbβ3 (JON/A-PE, Emfret Analytics, Würzburg, Germany) and P-selectin (anti-P-selectin-FITC, Emfret Analytics, Germany) for 15 min at RT. Samples were analyzed on an FACSCelesta.

### 2.3. Platelet Preparation

Mouse blood was collected from the retro-orbital plexus in heparin (20 U/mL) in TBS and centrifuged twice at 80× *g* for 7 min at RT to obtain the platelet-rich plasma (PRP). Consequently, PRP was supplemented with apyrase (0.02 U/mL) and prostaglandin (PGI_2_; 0.5 µM) and centrifuged at 640× *g* for 5 min. The pellet was resuspended in 1 mL Tyrode’s buffer (2.9 mM KCl, 134 mM NaCl, 0.34 mM Na_2_HPO_4_, 5 mM HEPES, 12 mM NaHCO_3_, 1 mM MgCl_2_, 5 mM glucose, and 0.35% BSA; pH 7.4) with 0.02 U/mL apyrase and 0.5 µM PGI_2_ and centrifuged twice at 640× *g* for 5 min. After adjustment of the platelet count, the washed platelets were supplemented with apyrase (0.02 U/mL) and rested for 30 min at 37 °C. To determine platelet size and count, whole blood was collected in EDTA-coated tubes and analyzed using a scil Vet abc Plus + Hematology analyzer.

### 2.4. Immunoblotting

Platelets were lysed and proteins were first separated by sodium dodecyl sulfate–polyacrylamide gel electrophoresis and then blotted onto polyvinylidene difluoride membranes. After blocking, polyvinylidene difluoride membranes were incubated with an anti-CYFIP1 (Millipore #2703674, Darmstadt, Germany), anti-FAM49b (SantaCruz #sc-390478, Dallas, TX, USA), anti-WAVE2 (Cell Signalling #3659, Waltham, MA, USA), anti-ARPC2 (Millipore #07-227), and/or an anti-α-Tubulin antibody (SantaCruz #sc-32293) overnight. Bands were visualized using horseradish peroxidase-conjugated secondary antibodies and enhanced chemiluminescence solution (MoBiTec, Goettingen, Germany). Images were taken using an Amersham Imager 600 (GE Healthcare, Chicago, IL, USA).

### 2.5. Platelet Spreading

For platelet-spreading experiments, coverslips were incubated with human fibrinogen (100 μg/mL; Sigma, St. Louis, MO, USA) overnight at 4 °C and blocked with BSA (1%) at 37 °C for one hour. The coated slides were washed with Tyrode’s buffer before preactivated platelets (0.01 U/mL thrombin (Roche, Basel, Switzerland)) were allowed to spread on the coverslips. At indicated time points, platelets were fixed with 4% paraformaldehyde (PFA) in PBS containing 0.1% Triton-X100 for 10 min, washed with PBS, and blocked with 5% BSA in PBS for one hour. Subsequently, the samples were stained with AlexaFluor 647-conjugated phalloidin for 1 h at RT, washed with PBS, and embedded in mounting medium. For analyses of platelet spreading phases, the cells were instead stained with Membright-488 (Idylle) diluted at 1:2000 in PBS for 5 min. Platelet spreading on micropatterns (see Section 2.8) was performed as described above, except that the platelets were given 40 min to spread before fixation. Spread platelets were visualized with a Leica TCS SP8 inverted confocal microscope (100×/1.4 oil objective, Leica Biosystems, Wetzlar, Germany). Images were analyzed using ImageJ 1.54h, National Institutes of Health, Bethesda, MD, USA.

### 2.6. Immunostaining of WAVE2

Eight-well chambered cover glasses (Sarstedt, Numbrecht, Germany) were coated with human fibrinogen (100 μg/mL; Sigma) diluted in PBS overnight at 4 °C and blocked with 1% BSA in PBS at 37 °C for one hour. The coated chambers were rinsed with Tyrode’s buffer before washed platelets, preactivated with 0.01 U/mL thrombin (Roche), were allowed to spread on the coverslips for 30 min. Then, the chambers were washed with PBS and the platelets were fixed with 4% PFA in PBS containing 0.1% Triton-X100 for 15 min. Fixed platelets were washed with PBS and blocked for 1 h with PBS containing 2.5% goat serum and 5% BSA. Subsequently, the samples were immunolabeled for 1 h with monoclonal rabbit anti-WAVE2 antibody (#3659, Cell Signaling) diluted at 1:200 in PBS containing 5% BSA. Following immunolabeling, the samples were washed 3 times with PBS and then stained with CF568-conjugated goat anti-rabbit antibodies (Biotium, #00044, Fremont, CA, USA), diluted 1:400 in PBS for 1 h. The cells were then washed 3 times with PBS and incubated overnight at 4 °C in phalloidin-conjugated AlexaFluor 647 (A22287, Life Technologies, Waltham, MA, USA), diluted 1:400 in PBS, and washed once with PBS before confocal or super resolution imaging.

*d*STORM: Reversible photoswitching of the dyes AlexaFluor 647 and CF568 was performed in a photoswitching buffer containing 100 mM β-mercaptoethylamine (Sigma) in PBS at pH ~7.4 in 8-well chambered cover glasses (Sarstedt). For a detailed description of the *d*STORM setup, see reference [22]. Imaging was performed with EMCCD cameras (iXon Ultra 897, Andor, Belfast, Northern Ireland) at a frame rate of 50 Hz for 30,000 (AlexaFluor 647) or 15,000 (CF568) frames. Image reconstruction was performed with the open-source software *rapid*STORM 3.3 [23]. Image processing was performed with ImageJ 1.54h, National Institutes of Health, Bethesda, MD, USA.

### 2.7. Transmission Electron Microscopy

Isolated platelets were fixed with 2.5% glutaraldehyde in 50 mM cacodylate buffer (pH 7.2). After embedding in epon 812, ultra-thin sections were generated and subsequently stained with 2% uranyl acetate and lead citrate. Sample visualization was performed with a JEOL JEM-2100 microscope. The platelet cytoskeleton of spread mouse platelets on human fibrinogen was visualized by platinum replica electron microscopy (PREM). The cells were washed for 5 min in PHEM with 0.75% Triton X-100, 1 µM phallacidin, 1 µM paclitaxel, and 0.1% glutaraldehyde. Subsequently, samples were washed in PHEM, with 0.1 µM phallacidin and 0.1 µM paclitaxel. The cells were fixed in PHEM with 0.1 µM phallacidin, 0.1 µM paclitaxel, and 1% glutaraldehyde for 15 min, and were finally washed twice with filtered dH_2_0. Subsequently, cells were treated with 0.1% tannic acid and 0.2% uranyl acetate, and dehydration was conducted in acetone. Critical point drying was performed in a Leica EM CPD300. Samples were coated with 1.2 nm of platinum with rotation at 45 °C and 3 nm of carbon at 90 °C without rotation under a high vacuum in a Leica EM ACE600. Finally, replicas were floated, picked up on formvar-carbon-coated grids and analyzed using a JEOL JEM-2100.

### 2.8. Micropattern Generation

Glass coverslips were exposed to an air plasma (0.2 mbar, ZEPTO, Diener) for 1 min, coated with 0.1% poly-L-lysine (PLL) for 30 min, washed 5 times with ddH_2_O, and let to dry. The dried cover slides were passivated with freshly prepared 100 mM HEPES buffer (pH 8.2) containing 100 µg/mL mPEG-SVA (Laysan Bio, Arab, AL, USA) and stored overnight at 4 °C. Residual mPEG-SVA solution was removed by washing the slides in ddH_2_O, and the slides were let to dry. Next, a polydimethylsiloxane stencil (Alvéole) was placed on the dried cover slides and 2.8 μL of a photosensitizer solution (consisting of 0.8 µL Product of Liaison for Protein Patterning (PLPP, Alvéole) and 2 μL MQ-H_2_O) were pipetted into each stencil well. The cover slides were then dried on a heat plate for 8 min at 50 °C. Micropatterns were introduced via maskless photopatterning (PRIMO, Alvéole) with a UV laser power of 30 mJ/mm^2^. After washing with ddH_2_O and PBS, the micropatterns were coated with AlexaFluor 546-labeled human fibrinogen (Invitrogen, Thermo Fisher Scientific, Waltham, MA, USA, 100 μg/mL in PBS) for 5 min and washed 4× with PBS. Slides were stored at 4 °C in humidified conditions until use.

### 2.9. Migration Assay

Murine blood was collected in acid–citrate–dextrose (ACD) solution (1:10) using nonheparinized capillaries. ACD (1:7) and Miakawa buffer (pH 6.5, 1:2) were added to the whole blood samples and centrifuged at 100× *g* for 10 min at RT. PRP was transferred into a fresh sample tube supplemented with Miakawa buffer (1:2) before centrifugation (5 min, 1200× *g*, RT). Supernatant was discarded and platelet count was adjusted to 400,000 platelets/μL in Miakawa buffer. Glass coverslips (24 × 24 mm) were acid-washed with 20% HNO_3_ for 1 h at RT, then washed with ddH_2_O for 15 min. Using a spin-coater, the coverslips were dried and subsequently silanized with hexamethyldisilazane. Migration chambers were created by cutting bottomless self-adhesive 6-channel slides (sticky-Slide VI 0.4, ibidi) into single channels and mounting the cut slides onto the coated coverslips. Channels were incubated with Miakawa buffer (pH 7.4) containing 0.2% human serum albumin and 37.5 mg/mL AlexaFluor 488-labeled human fibrinogen (Invitrogen, Thermo Fisher Scientific), then incubated for 10 min in the dark before being washed five times with Miakawa buffer (pH 7.4). A final concentration of 16,500 platelets/μL was added to a migration buffer consisting of 0.003% casein, 200 µM calcium chloride, 4 µM ADP, and 2 µM U46619 in Miakawa buffer (pH 7.4) and immediately pipetted into the migration chamber. Cells were incubated for 3 h at 37 °C in the dark before imaging on an inverted fluorescence microscope (Thunder Imager, Leica Biosystems, Wetzlar, Germany).

### 2.10. Whole-Blood Perfusion Assay

Glass cover slides were coated with 200 µg/mL Horm collagen at 37 °C overnight and washed with PBS before blocking for 1 h at 37 °C with PBS containing 1% BSA. Murine blood was collected in 20 U/mL heparin and consequently diluted at 2:1 in Tyrode’s buffer containing 2 mM Ca^2+^. Before the perfusion, the blood was incubated with 0.2 µg/mL Dylight-488-conjugated anti-GPIX antibodies at 37 °C for 5 min. The coated cover slides were placed in transparent flow chambers with a slit depth of 50 µm. The flow chambers were then connected to the mouse-blood-containing syringe. Perfusion was performed at a wall shear rate of 1000 s^−1^ for 4 min over the collagen-coated cover slides followed by a 4 min wash with Tyrode’s buffer at a shear rate of 1000 s^−1^. Image analysis was performed with ImageJ.

### 2.11. Data Analysis

Data are shown as mean ± standard deviation (s.d.) from ≥2 independent experiments per group unless otherwise stated. Differences were statistically analyzed using either the Mann–Whitney U-test or the *t*-test. Asterisks indicate * *p* ≤ 0.05; ** *p* ≤ 0.01; *** *p* ≤ 0.001. *p* values > 0.05 were rated as not significant.

## 3. Results

### 3.1. Cyfip1^−/−^, Fam49b^−/−^, and Cyfip1/Fam49b^−/−^ Mice Display Normal Platelet Count and Activation

**Figure 1:**
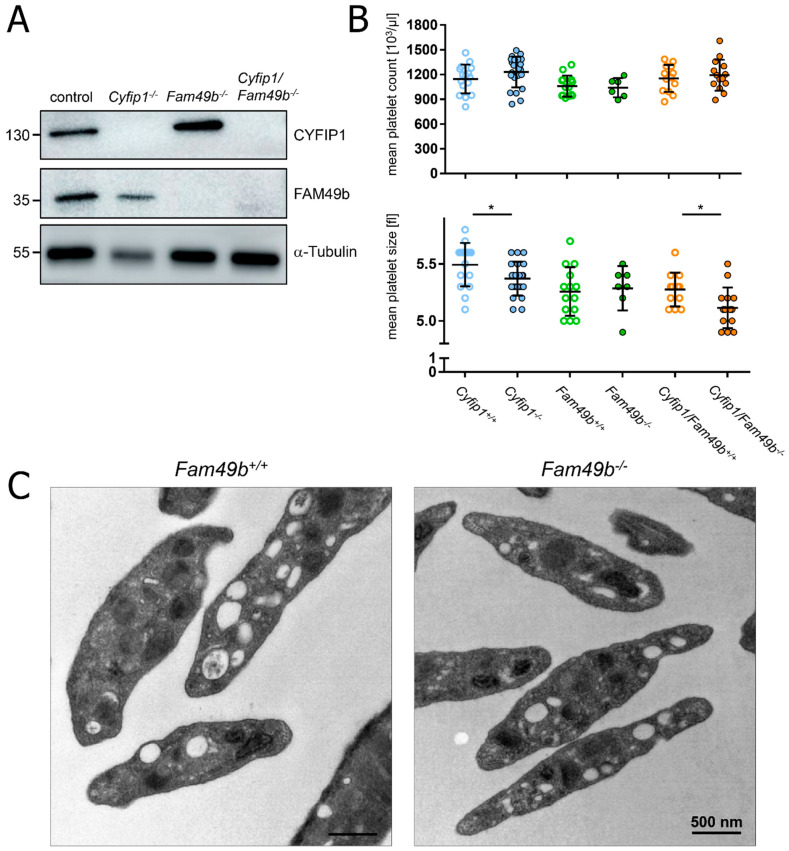
Platelet biogenesis of *Cyfip1*^−/−^, *Fam49b*^−/−^, and *Cyfip1*/*Fam49b*^−/−^ mice. (**A**) Expression of the indicated proteins in control and mutant platelets was assessed by Western blot analysis with α-Tubulin serving as loading control (n = 3). (**B**) Platelet count per microliter and platelet size, assessed by hematology analyzer (n = 7–17). Values are presented as mean plus or minus standard deviation (SD). Asterisks indicate * *p* ≤ 0.05. *p* values > 0.05 were rated as not significant. (**C**) Transmission electron microscopy of *Fam49b*^−/−^-platelets reveals no obvious differences in the platelet ultrastructure compared to control cells.

**Figure 2:**
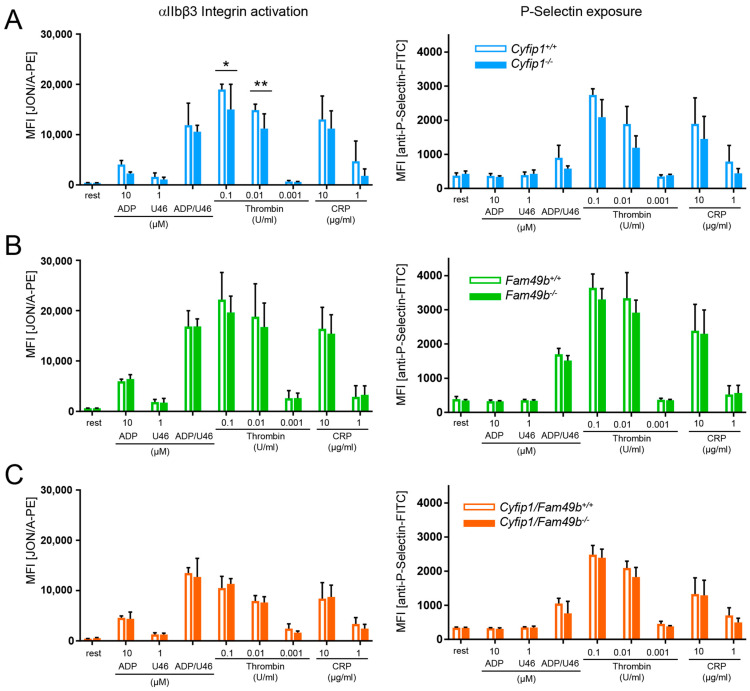
Unaltered activation of *Fam49b*^−/−^ platelets. Flow cytometric analysis of platelets lacking (**A**) CYFIP1, (**B**) FAM49b, and (**C**) CYFIP1/FAM49b. Cells were assessed for inside-out activation of the αIIbβ3 integrin (JON/A-phycoerythrin (PE) antibody) and degranulation-dependent P-selectin exposure (fluorescein isothiocyanate (FITC)-labeled anti–P-selectin antibody) in response to the agonists ADP, U46619 (thromboxane analog), thrombin, and CRP (n = 6). Values are presented as mean plus or minus SD; * *p* < 0.05, ** *p* < 0.01.

We confirmed the lack of CYFIP1 and/or FAM49b proteins in platelet lysates of *Cyfip1*^−/−^, *Fam49b*^−/−^, and *Cyfip1*/*Fam49b*^−/−^ mice by Western blot analysis (Figure 1A). All mice displayed a normal platelet count, with only a slight but significant reduction in platelet volume observed in those mice lacking CYFIP1 (Figure 1B). Platelet ultrastructure and expression of prominent glycoproteins were comparable between *Fam49b*^+/+^ and *Fam49b*^−/−^ mice (Figure 1C and Supplemental Table S1). We next assessed the activation of mutant platelets in response to various agonists as measured by αIIbβ3 integrin activation and P-selectin surface exposure. As was shown previously [10], *Cyfip1*^−/−^ platelets displayed only a slightly impaired activation (Figure 2A). However, the activation of *Fam49b*^−/−^ and *Cyfip1*/*Fam49b*^−/−^ platelets remained unaffected overall (Figure 2B,C). Altogether, these data indicate that FAM49b is dispensable for platelet biogenesis and agonist-induced platelet activation.

### 3.2. Cyfip1^−/−^ and Fam49b^−/−^ Platelets Spread Differentially on Fibrinogen-Coated Surfaces

**Figure 3:**
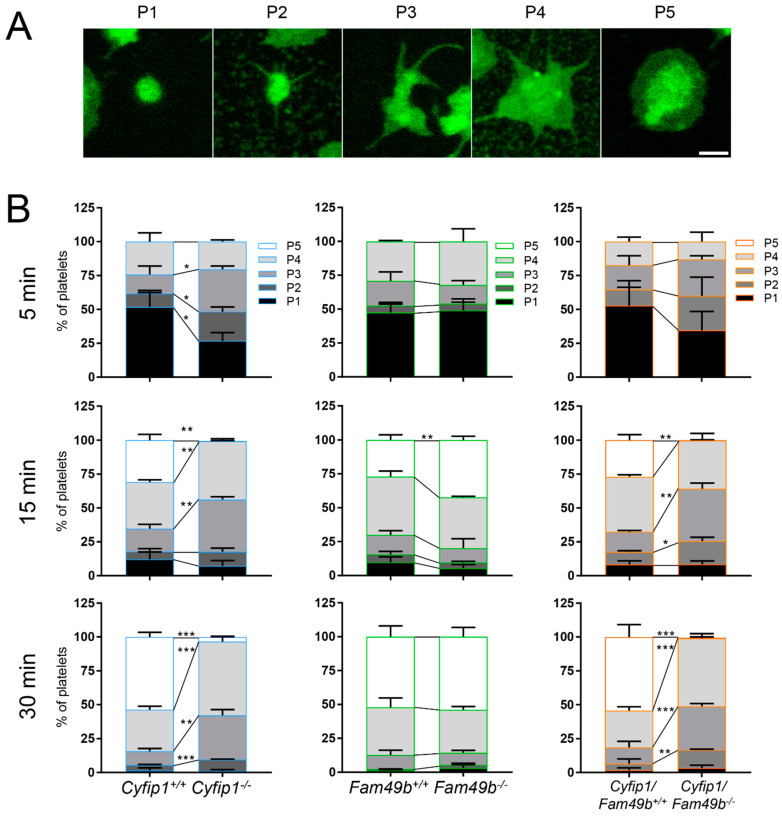
Differential spreading of isolated *Cyfip1*^−/−^-, *Fam49b*^−/−^-, and *Cyfip1*/*Fam49b*^−/−^-platelets on fibrinogen-coated surfaces. (**A**) Representative fluorescence images of different phases of spreading of wildtype platelets. Cell membranes were stained using Membright488. Scale bar: 1 µm. (**B**) Quantification of the different spreading phases of platelets fixed after 5, 15, and 30 min. Platelets mutated in *Cyfip1* were unable to reach spreading phase 5. Analyzed cells per genotype: n_5min_ > 200; n_15min_ > 400; n_30min_ > 700. Asterisks indicate * *p* ≤ 0.05; ** *p* ≤ 0.01; *** *p* ≤ 0.001. *p* values > 0.05 were rated as not significant.

It has been shown that the formation of platelet lamellipodia during spreading is critically dependent on the WAVE regulatory complex [10]. This prompted us to investigate the impact of FAM49b, a negative regulator of the WAVE regulatory complex [12], on platelet spreading. We therefore divided the process of platelet spreading into five consecutive stages (Figure 3A): (P1) adhesion of resting platelets, (P2) formation of filopodia, (P3) a combination of filopodia and premature lamellipodia-like structures, (P4) premature lamellipodia-like structures, and (P5) only lamellipodia. All mutant platelets were able to adhere to fibrinogen under static conditions (Figure 3B). However, as was shown previously [10], *Cyfip1^−^*^/*−*^ platelets were unable to form lamellipodia, and, thus, only reached phase 4 of platelet spreading (Figure 3B, blue columns). In contrast, while *Fam49b*^−/−^ platelet spreading displayed no difference to controls at the 5 and 30 min time points, we found a significantly increased portion of phase 5 cells after 15 min (*Fam49b*^−/−^: 42.39% ± 2.79, control: 27.03% ± 3.85), suggesting a faster spreading of these platelets during the intermediate stage of spreading (Figure 3B, green columns). Remarkably, *Cyfip1*/*Fam49b*^−/−^ platelets displayed virtually the same spreading dynamics as *Cyfip1^−^*^/*−*^ platelets at all tested time points, suggesting that CYFIP1 outranks FAM49b in the regulation of the WAVE regulatory complex.

### 3.3. Spread Fam49b^−/−^ Platelets Display a Reduced Circularity

**Figure 4:**
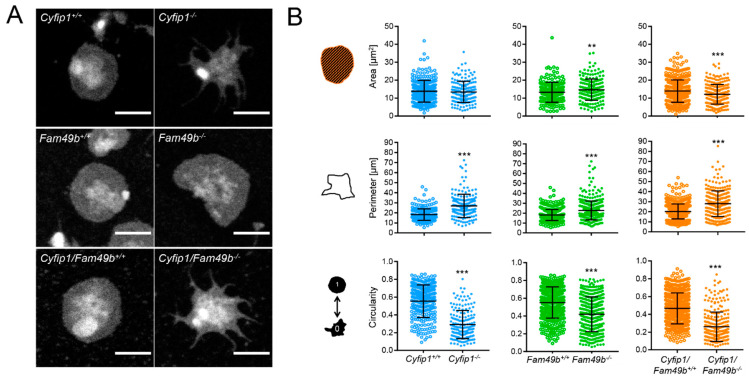

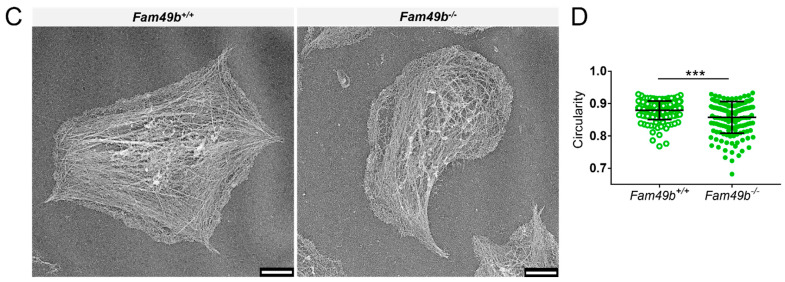
Morphology of spread *Cyfip1*^−/−^-, *Fam49b*^−/−^-, and *Cyfip1*/*Fam49b*^−/−^-platelets. (**A**) Representative confocal fluorescence images of mutant platelets after spreading on fibrinogen-coated surfaces for 30 min, compared to respective controls. In contrast to *Fam49b*^−/−^-platelets, cells mutated in *Cyfip1* displayed an abundance of filopodia and were unable to form lamellipodia. (**B**) Quantification of the cell area, perimeter, and circularity of all *Cyfip1*^−/−^-, *Fam49b*^−/−^-, and *Cyfip1*/*Fam49b*^−/−^ platelets present in a visual field after 30 min spreading, compared to respective controls. The perimeter of mutated cells was increased, whereas their circularity was significantly decreased. (**C**) Representative platinum replica electron microscopic images of *Fam49*^+/+^- and *Fam49b*^−/−^-platelets. (**D**) Circularity of *Fam49b*^−/−^-platelets in spreading phase 5 (Figure 3A). *Fam49b*^−/−^-platelets display a significantly reduced circularity compared to control cells. Scale bars: 2 µm in A; 900 nm in C. Values are mean plus or minus SD. ** *p* < 0.01 *** *p* < 0.001.

Next, to gain more insight into the role of FAM49b and its interplay with CYFIP1 in platelet spreading, we assessed the morphology of *Cyfip1*^−/−^, *Fam49b*^−/−^ and *Cyfip1*/*Fam49b*^−/−^ platelets spread on fibrinogen-coated surfaces. As shown in Figure 4A, both *Cyfip1^−^*^/*−*^ and *Cyfip1*/*Fam49b*^−/−^ platelets exhibited filopodia but lacked fully developed lamellipodia after 30 min of spreading on uniformly distributed fibrinogen. These qualitative observations are corroborated by our image-based quantifications of morphological characteristics, which show that CYFIP1-deficient platelets displayed a highly increased mean perimeter (Figure 4B, *Cyfip1*^+/+^: 18.26 µm ± 5.76, *Cyfip1^−^*^/*−*^: 26.91 µm ± 11.6; *Cyfip1*/*Fam49b*^+/+^: 20.29 µm ± 7.44, *Cyfip1*/*Fam49b*^−/−^: 27.98 µm ± 12.77) and only low circularity (*Cyfip1*^+/+^: 0.55 ± 0.18, *Cyfip1^−^*^/*−*^: 0.29 ± 0.16; *Cyfip1*/*Fam49b*^+/+^: 0.47 ± 0.17, *Cyfip1*/*Fam49b*^−/−^: 0.26 ± 0.17). In contrast, the morphology of *Fam49b*^−/−^ platelets was characterized by a large lamellipodium, similar to control cells (Figure 4A, second row). Both the mean projected cell area and perimeter were slightly but significantly increased in *Fam49b*^−/−^ platelets (14.66 µm^2^ ± 5.89; 22.69 µm ± 9.62), compared to controls (13.24 µm^2^ ± 5.56, 18.09 µm ± 5.54). In addition, our quantifications yielded a slightly decreased average circularity of *Fam49b*^−/−^ platelets (*Fam49b*^−/−^: 0.42 ± 0.19, *Fam49b*^+/+^: 0.55 ± 0.18). The increase of *Fam49b*^−/−^ cell area was also observed in platelets stimulated with ADP instead of thrombin immediately before being allowed to spread (Supplemental Figure S1). We next performed platinum replica electron microscopy to gain deeper insights into the cytoskeletal ultrastructure of spread *Fam49b*^−/−^ platelets (Figure 4C), yet we found no obvious difference in the nanoscopic organization of actin filaments compared to controls. To rule out that the reduced circularity of spread *Fam49b*^−/−^ platelets stems from the inclusion of the typically less circular phase 1–4 platelets, we then restricted our image-based morphology quantifications exclusively on phase 5 *Fam49b*^−/−^ platelets (Figure 4D). Interestingly, we still found that *Fam49b*^−/−^ platelets displayed a significantly decreased circularity, i.e., a higher polarity, as compared to controls. These results suggest that FAM49b plays a role in regulating the morphology of spread platelets.

### 3.4. Lack of CYFIP1 Facilitates Substrate Engagement on Anisotropically Distributed Fibrinogen

**Figure 5:**
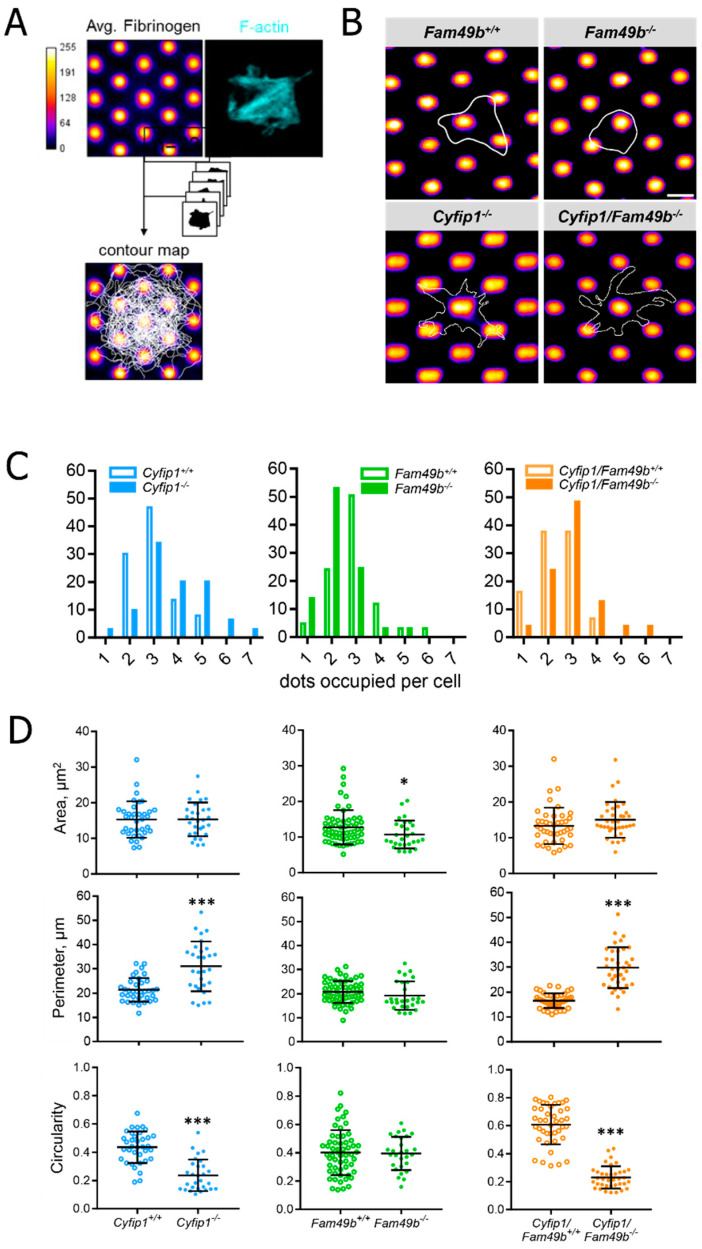
Morphology of *Cyfip1*^−/−^-, *Fam49b*^−/−^-, and *Cyfip1*/*Fam49b*^−/−^-platelets spreading on fibrinogen-coated micropatterns. (**A**) Platelets were allowed to spread on fibrinogen-labeled micropatterns for 40 min and stained for F-actin. (**B**) Representative cell contours of single *Fam49b*^+/+^, *Fam49b*^−/−^, *Cyfip1*^−/−^, and *Cyfip1*/*Fam49b*^−/−^ platelets used to quantify the number of dots occupied per cell. Dot diameter: 1.2 µm (SD: 0.1 µm); dot spacing: 1.9 µm (SD: 0.2 µm); Scale bar: 2.5 µm. (**C**) Quantification of occupied dots per platelet. *Cyfip1* mutant platelets occupied more dots compared to the respective control cells, whereas *Fam49b*^−/−^ platelets engaged fewer dots than controls. (**D**) Quantification of the cell area, perimeter, and circularity of *Cyfip1*^−/−^-, *Fam49b*^−/−^-, and *Cyfip1*/*Fam49b*^−/−^ platelets spread on fibrinogen-coated micropatterns. The area of *Fam49b*^−/−^ platelets was decreased, whereas *Cyfip1* mutant platelets displayed an increased perimeter and decreased circularity. Asterisks indicate * *p* ≤ 0.05; *** *p* ≤ 0.001. *p* values > 0.05 were rated as not significant.

To assess the morphology of spreading platelets under conditions closer resembling the in vivo platelet microenvironment, we generated surfaces with an anisotropic distribution of fibrinogen (2D structured micropatterns of fibrinogen) using a maskless photopatterning system (Figure 5A). A dotted structure was chosen to examine how platelet spreading is affected on minimally anisotropically distributed ligand spots. The dot size and distance were determined based on the resolution limit of the micropatterning system. Based on the cell contours (Figure 5B), we then quantified the number of dots occupied by each platelet as a measure of spreading efficiency. As seen in Figure 5C, the majority of control platelets occupied three dots. However, platelets lacking CYFIP1 were able to engage a higher number of fibrinogen-coated dots, with the majority of *Cyfip1*^−/−^ and *Cyfip1*/*Fam49b*^−/−^ cells occupying three up to seven dots (~70%, Figure 5C, blue and orange bars). This finding suggests that a platelet phenotype characterized by abundant filopodia formation might be advantageous for spreading on surfaces with anisotropic ligand density. In contrast, the majority of *Fam49b*^−/−^ platelets only connected to <3 dots (~68%, Figure 5C, green bars), indicating that the lack of negative WAVE regulation via FAM49b may be disadvantageous for platelet spreading on a nonuniformly coated fibrinogen surface. Interestingly, the cell area, perimeter, and circularity of *Cyfip1*^−/−^ and *Cyfip1*/*Fam49b*^−/−^ platelets spread on micropatterns closely resembled the respective parameters of *Cyfip1*^−/−^ and *Cyfip1*/*Fam49b*^−/−^ platelets spread on fully-coated fibrinogen surfaces (Figure 4B), indicating unimpeded platelet spreading when challenged by anisotropic ligand distribution.

### 3.5. Fam49^−/−^ Platelets Display Normal WAVE2 Localization

**Figure 6:**
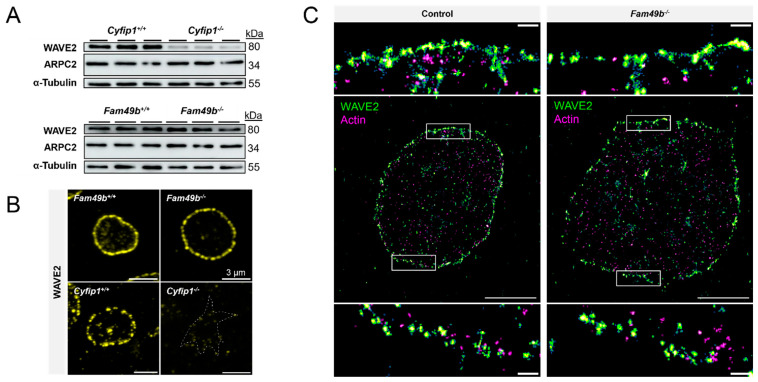
Localization of WAVE2 in *Fam49b*^−/−^ platelets. (**A**) Expression of WAVE2 and ARPC2 in control and mutant platelets was assessed by Western blot analysis. α-Tubulin served as loading control (n = 3). (**B**) Representative confocal fluorescence images of *Cyfip1*^−/−^ and *Fam49b*^−/−^-platelets stained for WAVE2. The dotted line in the *Cyfip1*^−/−^ image indicates the cell perimeter. (**C**) Super-resolved two-color *d*STORM images of WAVE2 (green) and F-actin (magenta) in control- and *Fam49b*^−/−^-platelets and magnifications of the boxed areas. Scale bars: 3 µm in (**B**); 2 µm in (**C**), 200 nm in magnifications of (**C**).

Loss of FAM49b was previously shown to increase RAC1-mediated localization of WAVE to the peripheral region of lamellipodia of fibroblasts [12]. Thus, we first probed the presence of WAVE2, i.e., the WAVE isoform mainly in complex with CYFIP1 [10], and the ARP2/3 subunit ARPC2, in platelet lysates of *Cyfip1*^−/−^ *and Fam49b*^−/−^ mice by Western blot analysis (Figure 6A). As also shown previously [10], we found that *Cyfip1*^−/−^ platelets displayed strongly reduced levels of the WAVE2 protein, indicating instability of the WAVE complex in absence of its subunit CYFIP1, while expression of ARPC2 remained unaltered. In contrast, in *Fam49b*^−/−^ platelets, neither WAVE2 nor ARPC2 expression showed any difference to controls.

We next investigated the subcellular localization of WAVE2 via confocal fluorescence microscopy. We found that WAVE2 localized along the cell perimeter in both *Fam49b*^+/+^ and *Fam49b*^−/−^ platelets spread on fibrinogen, consistent with the role of the WAVE complex in regulating lamellipodium formation (Figure 6B). However, unlike what was shown for FAM49b-deficient fibroblasts [12], we did not observe an obvious increase in WAVE2 localization in the lamellipodium. To exclude the possibility of nanoscopic differences in the peripheral WAVE localization, we employed diffraction-unlimited direct stochastic optical reconstruction microscopy of WAVE2 and F-actin (Figure 6C). However, while WAVE2 localized in close proximity to cortical actin filaments, we detected no obvious differences in the nanoscale WAVE2 distribution between *Fam49b*^+/+^ and *Fam49b*^−/−^ platelets.

### 3.6. Lack of FAM49b Does Not Affect Ex Vivo Thrombus Formation but Promotes Migratory Platelet Behavior

**Figure 7:**
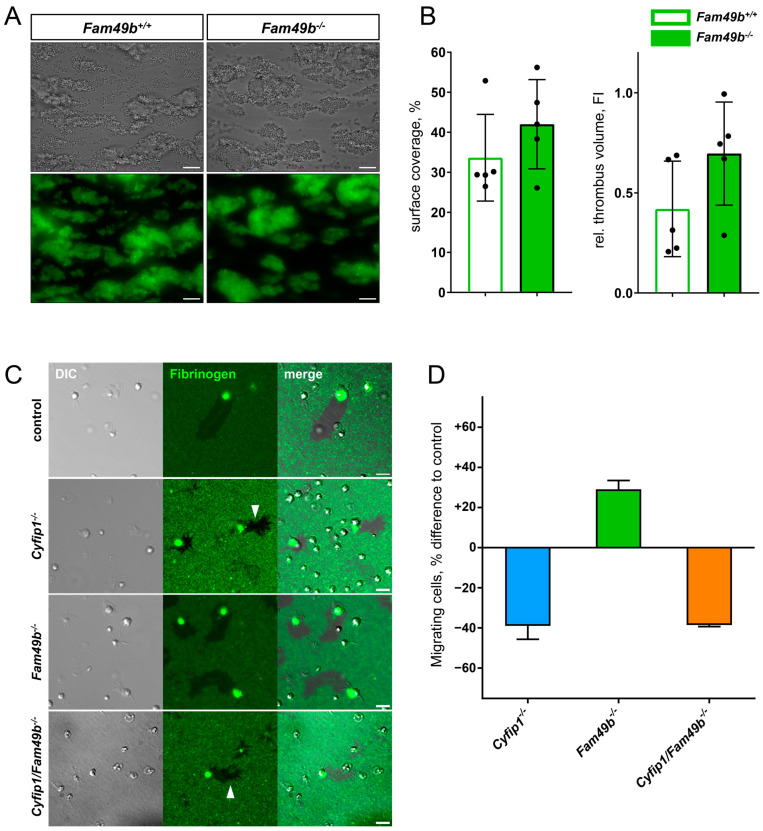
Thrombus formation and migration of mutant platelets. (**A**) Representative brightfield and fluorescence images of *Fam49b*^−/−^ platelets forming stable thrombi on collagen-coated surfaces at a wall shear rate of 1000 s^−1^. Scale bars: 20 µm. (**B**) Surface coverage and relative thrombus volume based on quantification of fluorescence signal (n = 5). (**C**) Representative differential interference contrast and fluorescence images of platelets migrating on fibrinogen-coated surfaces. The areas cleared by *Cyfip1* mutant platelets display a fuzzy perimeter (arrows). Scale bars: 5 µm. (**D**) Percentage difference of migrating cells compared to respective controls. The amount of migrating cells was increased in *Fam49b*^−/−^ platelets, whereas fewer *Cyfip1* mutant platelets migrated, compared to the respective controls (n = 3).

The lack of lamellipodia formation was shown to be dispensable for thrombus formation [10]. Next, we investigated whether the increased lamellipodia formation of *Fam49b*^−/−^ platelets affects thrombus growth. As seen in Figure 7A, both *Fam49b*^+/+^ and *Fam49b*^−/−^ platelets rapidly adhered to collagen and formed stable thrombi. Yet, neither the surface coverage nor the thrombus volume of *Fam49b*^−/−^ samples differed significantly from controls, indicating that thrombus formation is not altered by FAM49b deficiency (Figure 7B). However, lack of FAM49b was already reported to increase cell migration in other cell types [12], and downregulation of FAM49 was shown to enhance the invasiveness and migration of cancer cells [14,24]. However, the role of FAM49b in platelet migration is unclear. Thus, we assessed the migration (i.e., motility) of *Cyfip1*^−/−^, *Fam49b*^−/−^, and *Cyfip1*/*Fam49b*^−/−^ platelets on fibrinogen-coated surfaces. As seen in Figure 7C, the area cleared by migrating control and *Fam49b*^−/−^ platelets showed smooth edges, indicating lamellipodia-driven cell migration. In contrast, the areas cleared by *Cyfip1*^−/−^ and *Cyfip1*/*Fam49b*^−/−^ platelets displayed fuzzy edges (Figure 7C, arrows), consistent with cells primarily engaging their microenvironment via outgrowth of filopodia. These findings agree with our morphological analyses of platelets spread on fully-coated fibrinogen surfaces (Figure 4). To assess the migratory potential of the *Cyfip1*^−/−^, *Fam49b*^−/−^, and *Cyfip1*/*Fam49b*^−/−^ platelets, we then quantified the number of migrating cells in relation to all adherent, spread platelets compared to the percentage of migrating cells in the respective controls (Figure 7D). Interestingly, the percentage of migrating *Fam49b*^−/−^ platelets was increased by 29.08% (±4.35), whereas migration of *Cyfip1*^−/−^ and *Cyfip1*/*Fam49b*^−/−^ platelets was reduced by ~38% [11]. Thus, our analysis suggests that FAM49b negatively regulates platelet migration in the presence of a stable WAVE complex.

## 4. Discussion

This study provides the first description of the role of the FAM49b (CYRI-b) protein in mouse platelets. Our findings reveal that platelets lacking FAM49b exhibit enhanced spreading and larger lamellipodia formation on a uniformly fibrinogen-coated surface. In contrast, these mutant platelets displayed a reduced cell area when encountering a surface with patterned fibrinogen coating. FAM49b-deficient platelets often assumed a polarized shape (crescent shape) and were more primed to migrate in vitro. Platelets deficient for CYFIP1 and FAM49b were unable to form lamellipodia with the broad circular branched actin network, resembling the phenotype of the single CYFIP1-deficient platelets. These data show that the inhibitory effect of FAM49b on lamellipodia formation requires the presence of a functional WAVE complex.

Recently, we obtained a better understanding of how lamellipodial structures contribute to platelet function. It was demonstrated that the platelet lamellipodium is neither involved in the hemostatic function nor in stable thrombus formation ([10] and Figure 7A,B), but lamellipodia or even premature lamellipodial structures are required for platelet migration [11]. Yet, we still have gaps in our understanding of the proteins that control lamellipodia formation and, consequently, influence platelet migration. The results presented in our study suggest that FAM49b is a regulator of platelet morphology and migration. These findings are consistent with previous studies describing that FAM49b acts as a local RAC1 scavenger and thereby negatively regulates the WAVE complex [12]. Knockdown or knockout of FAM49b in COS-7 or CHL-1 cells promoted very large and broad lamellipodia and the WAVE2 protein was highly enriched in these cellular structures [12]. Similarly, we observed a faster transition of the FAM49b-deficient platelets to the lamellipodia formation phase. These data suggest that by removing the negative regulator of the RAC1-WAVE pathway in platelets, the kinetics of WAVE-driven lamellipodia formation is enhanced. Furthermore, we found an increase of their cell area after 30 min of spreading. However, the mutant platelets did not exhibit unusually large lamellipodia with increased WAVE2 localization, as previously observed for COS-7 and CHL-1 cells [12]. CHL-1 melanoma cells depleted of FAM49b frequently formed a crescent shape and those cells had a higher migration speed. In our study, platelets lacking FAM49b also showed a decrease in circularity, with a higher proportion adopting a crescent shape and displaying increased migration rate on fibrinogen. This indicates that the FAM49b protein acts as a negative regulator of platelet migration.

Previously, two distinct RAC1 pools were identified in platelets [25]. One RAC1 pool regulates actin dynamics involved in granule secretion, spreading, and clot retraction. The second RAC1 pool was found to control the signaling pathway downstream of the platelet-activating collagen receptor, glycoprotein VI [8,25]. To test a potential role of FAM49b in GPVI signaling, we performed a platelet activation assay and revealed that FAM49b-deficient platelets displayed comparable responses to all tested agonists, including the GPVI agonist CRP. Our data demonstrate that, unlike the small GTPase RAC1, FAM49b and CYFIP1 (ref [10] and in this study) are not essential for GPVI signaling.

Platelets lacking both FAM49b and CYFIP1 mirrored the phenotype of CYFIP1-deficient platelets. These mutant platelets were incapable of forming the characteristic orthogonally arrayed short actin filaments in the circumferential zone, a key structure typically observed in fully spread platelets. Instead, FAM49b/CYFIP1- and CYFIP1-deficient platelets only generated filopodial and premature lamellipodial-like extensions, which did not extend to the tips of adjacent filopodia [10,11]. Thus, the impaired migration of *Cyfip1*^−/−^ and *Cyfip1*/*Fam49b*^−/−^ platelets is likely connected to their inability to form mature lamellipodia. We observed that WAVE2 expression was reduced in CYFIP1 mutant platelets, indicating an instability of the WAVE complex in the absence of CYFIP1. Our findings in this study demonstrate that for FAM49b to exert its inhibitory effect on actin polymerization, the functional WAVE complex must be present.

The experiments in this study were exclusively conducted on mouse platelets. The genetic manipulation of the mouse genome has proven highly successful in elucidating protein functions in platelets. Mouse and human platelets exhibit a comparable cytoskeletal ultrastructure [3,26]. Additionally, it has been shown that the biophysical signatures between human and murine platelets are similar [27]. Moreover, mouse models, including the point-mutated *Myh9* mouse model [28], have been shown to mirror clinical manifestations observed in patients. Therefore, our findings concerning FAM49b in mouse platelets may indeed apply to human platelets as well.

## 5. Conclusions

This study revealed that FAM49b contributes to the cytoskeletal rearrangement during platelet spreading by counteracting WAVE-driven lamellipodium formation, e.g., restricting the projected cell area. However, this effect was only observed on uniformly fibrinogen-coated surfaces, but not on micropatterns with anisotropically distributed fibrinogen, which likely better reflects the microscale heterogeneity of ligand density in the extracellular matrix in vivo. Thus, while lamellipodia formation facilitates platelet migration, filopodial outgrowth allows for an increased engagement of the substrate in a lower-density environment. Hence, additional studies are needed to determine if the enhanced in vitro migration rate observed for FAM49b-deficient platelets translates to improved vascular surveillance function in mutant mice in vivo.

## Data Availability

The data generated and analyzed in this study are available from the corresponding authors on reasonable request.

## Supplementary Materials

The following supporting information can be downloaded at: https://www.mdpi.com/article/10.3390/cells13040299/s1, Figure S1: Quantification of the projected cell area of ADP-stimulated *Fam49b^+/+^-* and *Fam49b^−/−^*-platelets after 30 minutes of spreading on fibrinogen-coated surfaces; Table S1: Glycoprotein expression in *Cyfip1^−/−^*, *Fam49b^−/−^* and *Cyfip1/Fam49b^−/−^*platelets.
