# Supplementary material for: Differential Role of the RAC1-Binding Proteins FAM49b (CYRI-B) and CYFIP1 in Platelets
Source: Cells. 2024 Feb 6;13(4):299. doi: 10.3390/cells13040299 (PMC10886774; doi:10.3390/cells13040299)
Supplement: Supplementary file 1 [file cells-13-00299-s001.zip › cells-2801705-supplementary.pdf]

# SUPPLEMENTAL INFORMATION

# Differential role of the RAC1-binding proteins FAM49b (CYRI-B) and CYFIP1 in platelets

Dmitri Sisario[1], Markus Spindler[1], Katharina J. Ermer[1,†], Noah Grütz[1], Leo Nicolai[2,3], Florian Gaertner[2,3], Laura M. Machesky[4], Markus Bender[1,*]

[1]Institute of Experimental Biomedicine–Chair I, University Hospital Würzburg, 97080 Würzburg, Germany

[2]Medizinische Klinik und Poliklinik I, University Hospital Ludwig-Maximilian University, 81377 Munich, Germany

[3]German Centre for Cardiovascular Research, Partner Site Munich Heart Alliance, 81377 Munich, Germany

[4]Department of Biochemistry, University of Cambridge, Sanger Building, 80 Tennis Court Road, Cambridge CB2 1GA, UK

[†]Current affiliation: Comprehensive Heart Failure Center, Department of Translational Science, University Hospital Würzburg, 97078 Würzburg, Germany.

*Corresponding author:

Markus Bender, Email: bender_m1@ukw.de

**Supplemental Table S1**. Glycoprotein expression in *Cyfip1*$^{-/-}$, *Fam49b*$^{-/-}$ and *Cyfip1/ Fam49b* $^{-/-}$ platelets. Platelet glycoprotein expression was determined via flow cytometry (n = 6). Values are presented as mean ± standard deviation. *$P$ < .05, **$P$ < .01.

| | *Cyfip1*$^{+/+}$ | *Cyfip1*$^{-/-}$ | significance |
|---|---|---|---|
| β1 | 7044 ± 200,6 | 7125 ± 131,5 | ns |
| GPIb | 16577 ± 789,7 | 15988 ± 794,3 | ns |
| GPV | 11152 ± 337,5 | 10791 ± 353,8 | ns |
| GPIX | 20852 ± 440,2 | 19843 ± 875,1 | * |
| αIIbβ3 | 28755 ± 1116 | 27538 ± 1012 | ns |
| α2 | 1934 ± 61,17 | 1763 ± 238,1 | * |
| GPVI | 1973 ± 61,02 | 1918 ± 145 | ns |
| CLEC-2 | 5024 ± 327 | 4732 ± 502,2 | ns |

| | *Fam49b*$^{+/+}$ | *Fam49b*$^{-/-}$ | significance |
|---|---|---|---|
| β1 | 7552 ± 151,7 | 7245 ± 179 | ** |
| GPIb | 16033 ± 1886 | 17670 ± 979,7 | ns |
| GPV | 11086 ± 327,6 | 10993 ± 288,1 | ns |
| GPIX | 21730 ± 461,3 | 21062 ± 624,7 | ns |
| αIIbβ3 | 30189 ± 1816 | 27808 ± 1192 | ns |
| α2 | 2257 ± 95,67 | 2137 ± 116,5 | ns |
| GPVI | 2090 ± 95,9 | 2091 ± 47,71 | ns |
| CLEC-2 | 5503 ± 168,6 | 5461 ± 228,5 | ns |

| | *Cyfip1/Fam49b*$^{+/+}$ | *Cyfip1/Fam49b*$^{-/-}$ | significance |
|---|---|---|---|
| β1 | 6941 ± 163 | 7023 ± 143 | ns |
| GPIb | 14478 ± 466 | 14004 ± 490 | ns |
| GPV | 10877 ± 323 | 10487 ± 357 | ns |
| GPIX | 17821 ± 247 | 17172 ± 509 | * |
| αIIbβ3 | 18368 ± 322 | 17698 ± 610 | ns |
| α2 | 2668 ± 127 | 2689 ± 20,8 | ns |
| GPVI | 2486 ± 28,4 | 2474 ± 89,4 | ns |
| CLEC-2 | 5278 ± 213 | 5065 ± 223 | ns |

**Supplemental Methods**

Platelet spreading upon ADP-stimulation

For platelet spreading experiments, coverslips were incubated with human fibrinogen (100 µg/ml; Sigma) overnight at 4°C and blocked with BSA (1%) at 37°C for one hour. The coated slides were washed with Tyrode's buffer before preactivated platelets (10 µM ADP (Sigma)), were allowed to spread on the coverslips. After 30 minutes, platelets were fixed with 4% paraformaldehyde (PFA) in PBS containing 0.1% Triton-X100 for 10 min, washed with PBS and blocked with 5% BSA in PBS for one hour. Subsequently, the samples were stained with AlexaFluor 647-conjugated phalloidin for 1 hour at RT, washed with PBS and imbedded in mounting medium. Spread platelets were visualized with a Leica TCS SP8 inverted confocal microscope (100x/1.4 oil objective, Leica, Germany). Images were analyzed using ImageJ.

**Supplemental Figures**

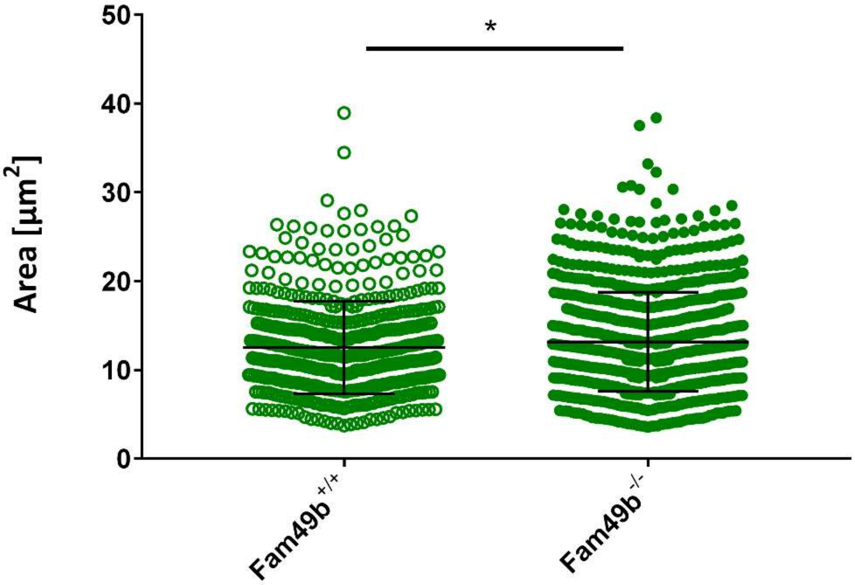


**Supplemental Figure S1.** Quantification of the projected cell area of ADP-stimulated *Fam49b*$^{+/+}$- and *Fam49b*$^{-/-}$-platelets after 30 minutes of spreading on fibrinogen-coated surfaces. The area of mutated cells was increased. For each condition, at least 500 cells were analyzed. Values are mean plus or minus SD. *$P < 0.05$.
